# Some Neuroses Associated with Menopause

**Published:** 1894-02

**Authors:** 


SOME NEUROSES ASSOCIATED WITH THE MENOPAUSE.—At a recent meeting of the Medical Society of London, Savage (*Brit. Med. Journal,* No. 1714, p. 995) pointed out that many mental disorders occur at the menopause that cannot be traced to this as a simple cause, though in many cases it is a predisposing cause. No special form of insanity can be called climacteric, but as a rule there is a tendency to mental depression, rather than to excitement. Any of the symptoms of the menopause may be so exaggerated as to assume serious mental aspects. For instance, there may be a kind of climacteric hypochondriasis, manifested by complaints referred to the circulation, to the head, to the skin, or to the viscera. The unnatural feelings in the skin or about the body may lead to false accusations, and the development of hair on the face may lead to miserable feelings of being unnatural and unwomanly. Deafness sometimes arises at the time of the menopause, and may lead to various delusions, particularly of a suspicious nature. There is a special form of insanity which is most frequent at the climacteric period, in which, with progressive deafness, there is a progressive development of delusions of suspicion, a form that is difficult of cure and which may be parallel to general paralysis of the insane, though affecting the sensory side and not so fatal as the disease affecting the more organic side of life. There is a great tendency to the development of disorders of the reproductive organs and of the feelings associated with their function. Thus, there is frequently jealousy, irritability, and general domestic incompatibility. There is also frequently hysterical disorder of one form or another; in single women there are false ideas as to marriage proposals and the like, and in married women a redevelopment of sexual desire that may lead to various forms of sexual excess. There is a great tendency to seek stimulants, morphine and similar danger. The prognosis is fairly good in many cases, though in a large proportion, especially among those that have had other attacks of insanity, recovery does not ensue. Rarely the climacteric period is attended with a relief from existing mental or nervous affections. General rather than special measures are indicated, and the promiscuous use of the bromides is to be deprecated.—*Med. Progress.*

